# Rhetorical strategies in Chinese and English talk show humor: a comparative analysis

**DOI:** 10.3389/fpsyg.2024.1380702

**Published:** 2024-05-09

**Authors:** Zhou Tianli, Shiyue Chen

**Affiliations:** ^1^School of Foreign Languages, Zunyi Medical University, Zunyi, Guizhou, China; ^2^Faculty of Modern Languages and Communication, Universiti Putra Malaysia, Serdang, Selangor, Malaysia

**Keywords:** rhetorical strategies, talk show, humor, comparative analysis, Chinese and English

## Abstract

Humor is a kind of cognitive psychology activity, and it is diverse among individuals. One of the main characteristics of talk shows is to produce humorous discourse to make the audience laugh; however, rare studies have made a deeper comparative investigation on the rhetorical strategies in different language humorous utterances. Therefore, the current study adopted a mixed method of sequential explanatory design to identify the types of rhetorical strategies in the monolog verbal humor of Chinese and English talk shows, examine their similarities and differences. Two hundred monolog samples from 2016 to 2022, which consisted of 100 monologs of Chinese talk shows (CTS) and 100 monologs of English talk shows (ETS), were downloaded from the internet as language corpus. Berger’s theory was adopted to identify the types of rhetorical strategies. Based on the obtained findings, this study found that both language talk show hosts use a variety type of rhetorical strategies to produce humorous discourse. The comparison of similarities and differences revealed that the most frequently used rhetorical strategies in both talk shows were almost similar (e.g., satire, exaggeration, facetiousness, and ridicule), but the percentage of usage of these various rhetorical strategies in both talk shows was slightly different. Interestingly, misunderstanding occurred 20 times in CTS but was not found in ETS. Meanwhile, simile and personification were used more often in ETS. Conclusively, this study contributes valuable insights on the use of different types of rhetorical strategies to create verbal humor in different language contexts.

## 1 Introduction

Walker (1998) defined humor as “the ability to smile and laugh, and to make others do so… humor takes many forms ranging from the casual level of the joke told to friends to the sophistication of a Shakespearean comedy” (p. 3). Wickberg (1998) regarded humor as a sympathetic form of amusement that was linked to pathos, and was distinguished from wit, which was perceived as more aggressive and less socially desirable. Furthermore, Carrell (2008) expressed the following: “For some, humor is its physical manifestation, laughter; for others, humor is the comic, the funny, or the ludicrous. For still others, humor is synonymous with wit or comedy. And so, the terminological fog abounds” (p. 3). Obviously, the sense of humor is a kind of cognitive psychology activity, and it is diverse among individuals. Many Linguists, psychologists, and anthropologists have taken humor to be an all-encompassing category, covering any event or object that elicits laughter, amuses, or is felt to be funny (Attardo, 1994).

Following the development and applications of rhetorical research, rhetoric has become an important research topic in the fields of politics, society, religion, and science; scholars and educators have expressed their research interest in rhetoric (Herrick, 2018). Booth (1988) confirmed the view that all verbal endeavors are under the control of rhetoric, and this includes logic, dialectic, grammar, philosophy, history, and poetry. McKeon (1989) expressed almost similar view—he believed that rhetoric is best understood as a universal architectural art. In other words, rhetoric is a master discipline that organizes and structures other arts and disciplines.

Scholars have emphasized the essential role of rhetorical strategies in humorous discourse—these strategies motivate humor and addresses specific communication purposes. Burke (1945) argued that there is “persuasion” wherever there is “meaning,” which evidently suggests the importance of rhetoric in human discourse and their humorous discourse. In the book of *Rhetoric*, Aristotle considered the practical utility of humor and believed that it must serve the orator’s logic (Attardo, 2010). At the structural or linguistic level, studies have demonstrated the importance of rhetoric in creating humorous discourse. A few prior studies viewed such humor as “rhetorical humor.” For instance, Berger (1976) proposed the use of 45 strategies among comedy writers and humorists to create humor. Most of these strategies, such as allusion, exaggeration, irony, and puns, are rhetorical in nature. Then Weaver (2015) proposed that rhetoric is “convincing communication,” as one of the fundamental rhetorical communication methods lies in the similarities between hilarious incongruity and metaphor. Weaver (2015) considered humor as a persuasive communication tool that can reduce people to absurdism, and it is the content of the premises that must be examined in order to determine where the rhetorical impact of humor lies.

Evidently, humor is one of the characteristics of a talk show, and it is a concentrated form of humor expression that can make the audience laugh due to its humorous language. Talk shows have gained growing popularity in the past decades (Feng, 2017) because talk shows offer information and entertainment. This has motivated growing research interest among linguists due to the rich humorous discourse in talk shows. Prior studies on the humor of talk shows mainly focused on humor discourse analysis (Hao, 2018), humor pragmatic strategies (Zhang, 2019), and humorous language production mechanisms (Zhou, 2015; Chen, 2019), but rhetorical strategies in talk shows related to humor production have remained underexplored.

Additionally, rhetorical strategies are generally complex across different language humorous discourses. English and Mandarin are two of the most broadly used main languages in the world (Cheung et al., 2004). English language belongs to Indo-European language family, whereas Chinese language belongs to Tibetan language family (Yitong, 2020). Therefore, both languages are different in shape, pronunciation, and way of use. These distinctions make it more challenging to comprehend their humorous discourse. However, English has recently received increasing attention in China, and it has progressively turned into a necessary instrument for Chinese people to communicate with English speakers. Talk shows that originated from the United States in the 1950s were imported to China during the mid-1990s and have been developed as one of the most popular entertainment programs in China (Geng, 2017). Moreover, CTS and ETS have the largest audience in the world due to the large population base in China and the widespread popularity and acceptance of English language. Based on the above reasons, a comparative study on CTS and ETS was deemed noteworthy for the current study to explore, particularly with the abundant humorous discourse resources available. Therefore, the present study attempted to explore and compare the choice of rhetorical strategies in the humorous discourse of Chinese talk shows and English talk shows. The specifical research objectives are two forward:

1. To identify the types of rhetorical strategies used to create humor in the monologs of CTS and ETS.
2. To compare the similarities and differences of rhetorical strategies used to create humor in CTS and ETS.

## 2 Literatures

### 2.1 Studies on rhetorical strategies

Aristotle (1954) defined the classical authority of rhetoric as “the function of finding a possible means of persuasion in any given situation” (p. 24), which shows that early rhetoric was aimed at persuading the audience; it is almost synonymous with “persuasion.” Aristotle (1954) also argued that “rhetoric is the counterpart of dialectic” (p. 25), implying a characteristic of rhetoric—unlike dialectic, rhetoric is not substantive because it has no subject of its own but plays a substantial role in other disciplines, which makes it cross-disciplinary in nature. Basically, rhetoric is something that carries too much content but has no substantial subject matter. So, scholars examined rhetorical strategies with different rhetorical theories and perspectives.

According to Aristotle (2007), the use of rhetorical tactics involves the deliberate use of three persuasive techniques, which are expertly organized and articulated within a speech or written composition: ethos, pathos, and logos (Aristotle and Roberts, 2004). Ethos denotes the speaker’s personality needs to demonstrate good character and credibility. Pathos refers to evoking the right emotions in the audience, while logos means providing logically reasoned arguments (Wachsmuth et al., 2018). Then, numerous prior studies adopted Aristotle’s three models of persuasion as rhetorical strategies to analyze different discourses and speeches. For instance, Troje (2018) analyzed the application of rhetorical strategies by the supporters of social procurement in Sweden and revealed the use of various rhetorical strategies, including *ethos*, *logos*, *pathos*, and arguments, to persuade and appeal to the potential supporters’ emotions. The study provided an overview of many types of rhetorical arguments for those with the intention to make social procurement. In a more recent study, Liu et al. (2019) performed text analysis of the rhetorical strategies applied by CEOs and firms’ corporate social performance (CSP). Based on Aristotle’s *pathos*, *ethos*, and *logos* strategies, the study examined whether CEOs applied rhetorical strategies and the influence of rhetorical strategies on CSP. Certain studies described rhetorical strategies as a set of mechanisms that organize language to shape people’s understanding of technologies and managerial practices (Heracleous and Barrett, 2001; Suddaby and Greenwood, 2005; Brown et al., 2012).

According to Bernardi (2017), rhetorical strategies help organizational actors to negotiate and transform policies. There is the Fairclough’s typology (2003, p. 41–42) to explore rhetorical strategies within different contexts. Fairclough’s (2003) typology includes openness (accept differences), polemic (accentuate difference and conflict by meaning, norms, and power), resolution (try to find out ways to solve differences), bracketing (set aside differences and opt to focus solely on the things in common), and normalization (reach commences and agreement by overcoming differences and surpassing the different norms) (Bernardi, 2017). Besides that, the Create a Research Space (CARS) model, which was designed by Swales (1990), has been used to explain rhetorical strategies. Accordingly, the model consists of three “moves”: (1) establishing a territory; (2) establishing a niche; (3) occupying the niche. Swales (2004, p. 228) defined a “move” as “a discoursal or rhetorical unit that performs a coherent communicative function in a written or spoken discourse.” In particular, “establishing a territory” outlines the existing information of the topic, “establishing a niche” seeks knowledge gap, and “occupying the niche” demonstrates the relevance of the study (Marta, 2019). Furthermore, there have been several studies on a wide range of discourses through the lenses of phonological rhetorical strategies, lexical rhetorical strategies, and grammatical rhetorical strategies (Song, 2005; Fang, 2011; Bu, 2014), while Aytan et al. (2021) explored the intricacies of how English euphemisms and dysphemism serve as rhetorical and strategic tools in political media discourse.

Conclusively, these prior studies expanded the connotation and outer edge of rhetorical strategies, the current study regarded that rhetorical strategies encompasses all linguistic rhetorical devices to achieve communicative goals. Many prior studies analyzed discourse in terms of Aristotle’s rhetorical strategies (i.e., *pathos*, *ethos*, and *logos*) but did not specifically address the rhetorical strategies achieved through which rhetorical devices. Aristotle’s rhetorical strategies have been widely used to persuade the audience to gain their support and may be applicable to any context, but it does not necessarily link to humorous discourse, which was the main feature of the current study. Other rhetorical theories that some previous study adopted also applied under some certain humorous context. While the current study focused on the rhetorical devices in humorous discourse and explored the similarities and differences of the rhetorical strategies used in the humorous discourse of CTS and ETS. Hence, a modified rhetorical strategy list based on Berger’s (1976) humor typology was adopted including rhetorical strategies of allusion, analogy, bombast, definition, exaggeration, facetiousness, insults, infantilism, irony, misunderstanding, metaphor, over-literalness, puns, personification, repartee, ridicule, sarcasm, satire, and simile. Besides that, previous studies analyzed rhetorical strategies from a macro-perspective, but the current study had its own characteristics, specifically this study compared rhetorical strategies from a micro-perspective.

### 2.2 Studies on humor and rhetoric

Verbal humor comprises a diverse array of linguistic humor phenomena. Studies have examined rhetorical strategies in humorous discourse from a variety of perspectives.

Firstly, prior studies explored the relationship between rhetorical strategies and humor and their role in creating humor. As early as 1991, Attardo and Raskin pointed out that metaphors are particularly well-suited to express hilarious intent, satisfying all the prerequisites for comedy outlined in the general theory of verbal humor. Weaver (2015) proposed that rhetoric is one of the fundamental rhetorical communication methods lies in the similarities between hilarious incongruity and metaphor. Wang (2019) contended that using linguistic rhetorical strategies to create humor is important, which is consistent with the views of Weaver (2012, 2015). Moreover, Weaver (2015) agreed with Palmer (1987) on his view that comic is strongly rooted in the area of rhetoric due to the structural link between metaphor and humor—the comparable semantic structure. Similarly, Piata (2016) stated that the relationship between metaphor and humor has long been thought as a conceptual similarity—in that both phenomena deal with duality in diverse ways. Piata (2016) attempted to provide a new perspective on this topic by arguing that metaphor and humor, rather than diverging, converge in terms of their evaluative function in language. Besides that, Droog and Burgers (2020) introduced a typology named “humoristic metaphors in satirical news” (HMSN) to demonstrate how satirists potentially use metaphors to materialize and switch among four different discourse modes. The study then concluded that practically all metaphors employed in satirical news possess humorous rhetorical goal since the principal purpose of satire is to make people laugh. Furthermore, in 2023, they link the HMSN typology with the General Theory of Verbal Humor (GTVH) to deepen the knowledge of how metaphorical humor is employed in satirical news to explain or criticize the current affairs. According to the GTVH, any verbal humor needs to reference six interconnected Knowledge Resources (KRs) (script-opposition, logical mechanism, situation, target, narrative strategy, and language) (Attardo and Raskin, 1991). Their study reveals that some KRs can support metaphorical jokes in achieving their communicative function(s); however, certain KRs limit the ways in which other KRs or specific communicative function(s) can be expressed (Droog and Burgers, 2023).

Meanwhile, Veale (2013) emphasized that humorous descriptions are disguised as similes at times—many structural and semantic features that reflect the characteristic of poetic similes are present in humorous similes although none of those appear to be necessary or sufficient to make a simile creative and humorously creative, but many often mark irony or ridicule with a semantic imprecision marker, such as “about.” Therefore, Veale (2013) argued that humorous similes exhibit all the hallmarks of verbal humor, from language ambiguity to expectation violation and suitable incongruity—this proves the critical role of similes in humor. Tabacaru and Feyaerts (2016) attempted to clarify the ability of metonymy to produce humorous impact by stretching its various layers of meaning to justify its power to produce humor. In a more recent study, Godioli and Little (2022) found that satire and exaggeration are often expressed together in courts to produce humor.

Secondly, verbal humor is commonly expressed via rhetorical strategies (Mulyadi et al., 2021). It may take the form of irony, sarcasm, ridicule, puns, or other rhetorical strategies applicable to the situation (Mulyadi et al., 2021). Prior studies examined various rhetorical strategies in conjunction with various humorous contexts to explain the functions of rhetorical humor, such as jokes (Juckel et al., 2016; Heidari-Shahreza, 2017; Yahiaoui et al., 2019) and political discourse (Orkibi, 2016; Piata, 2016; Ryabova, 2021). Rochmawati (2017) examined the pragmatic and rhetorical strategies in English-written jokes. The study argued that written English is significantly different from the verbal English—the former is more complex and has more grammatical complexity. Focusing on the humor mechanism of written jokes from the pragmatic and rhetorical perspectives, the study demonstrated the use of rhetorical and pragmatic strategies in written jokes for rhetorical goals and humor-related functions. A few other studies studied rhetoric in political elections and movements’ discourses. Since rhetoric is a powerful tool in persuasion, studies have argued the use of rhetorical humor in political movements against powerful institutions, social hegemonies, political elites and performance to achieve their goals (Orkibi, 2016; Laaksonen et al., 2022). Additionally, candidates often use rhetorical humor to persuade people to vote for them in the presidential election. There have been various studies on rhetorical humors in different situations, such as the function of ironic humor when it comes to crisis communication (Vigsø, 2013) and the function of rhetorical humor in decorum (Waisanen, 2015). Moreover, Stoyanova (2021) examined metaphor as a means of creating humorous effect in media texts. The role of rhetorical strategies varies across distinct categories of humor contexts, contingent upon the speaker’s intended message in particular circumstances (Tianli et al., 2022).

Previous studies presented robust evidence on the essential role of rhetorical strategies in humorous discourse—these strategies motivate humor and addresses specific communication purposes. According to Rutter (2001), the analysis of rhetorical strategies in stand-up comedy performance revealed the strong relationship between the use of rhetorical strategies and audiences’ laughter. The study further suggested that rhetorical humor is achieved through the rhetorical use of language. Dedace et al. (2023) explored the humor and language of Filipino comedians revealed that the unique rhetorical strategies they use in making humor are influenced by comedians’ culture. Besides that, Heidari-Shahreza (2017) pointed out the use of various rhetorical devices by Persian comedians to signal the appropriate time for the audience to respond, particularly to laugh. Rochmawati (2017) examined rhetorical strategies in written jokes and noted the recent shift in the field of humor research, emphasizing linguistic humor and involving the field of rhetoric—this established a trend for future humor research.

Overall, previous studies expanded the existing literature of this linguistic phenomenon of humor and presented deeper understanding of rhetorical strategies in humorous discourses. However, rhetorical strategies in the humorous discourse of CTS and ETS have not been explored. To response the gap, focusing on the rhetorical humor in TV talk shows, the current study performed comparative analysis of CTS and ETS, which was different from the prior studies.

## 3 Methodology

A mixed method of sequential explanatory design was adopted in this study. The mixed-methods sequential explanatory design is a research approach that involves two different phases: quantitative followed by qualitative (Creswell, 2003). Initially, the researcher collects and analyses the quantitative data, which typically involves numeric information. The second phase involves collecting and analyzing qualitative data, usually in the form of text, which helps to explain or elaborate on the quantitative results obtained in the first phase. The two phases are connected in the intermediate stage of the study, and the second qualitative phase builds on the first quantitative phase (Ivankova et al., 2006). The rationale for using this method in this study is that quantitative data and subsequent analysis provide a broad understanding of the research problem, whereas qualitative data and analysis refine and explain those statistical results by delving deeper into the factors and interpreting the results and examples (Creswell, 2003).

### 3.1 Data sources

CTS and ETS represented this study’s data sources. In particular, three American English talk shows and three Chinese Mandarin talk shows were selected. The reasons for selecting American English talk shows and Chinese Mandarin talk shows as data sources were threefold. Firstly, as the birthplace of talk shows, the United States has a long history of talk shows and a diverse range of talk show programs (Timberg and Erler, 2002), offering the possibility of more sample choices for the current study. Secondly, the United States is a relatively young country with strong cultural inclusiveness, which is particularly suitable for the development of talk shows and creates good environment for talk shows. Therefore, American English talk shows have led the development of the global trend of talk show programs, which provided a relatively new and valuable corpus for the current study. Thirdly, Chinese Mandarin talk shows have been adapted by American talk shows, and over the years, Chinese Mandarin talk shows have made new developments in program innovation through the incorporation of the premise of Chinese local culture (Geng, 2017). The contrasting features of this study were better realized through the comparison of humorous discourse of CTS with that of ETS in terms of similarities and differences.

There were specific selection criteria of data sources in this study. Firstly, talk shows must contain a monolog section. Secondly, ETS should use standard English, while CTS must use Mandarin, instead of dialects. Table 1 presents the details of the selected data sources.

**Table 1:** Data sources.

Talk show	Program	Talk show hosts/comedians	Download platform
CTS	*Tonight 80’s Talk Show*	Wang Zijian	Tencent
	*Jin Xing Show*	Jin Xing	Tencent
	*Rock and Roast (Talk Show Conference)*	Fourteen comedians	Tencent
ETS	*The Ellen DeGeneres Show*	Ellen DeGeneres	YouTube
	*The Tonight Show Starring Jimmy Fallon*	Jimmy Fallon	YouTube
	*The Daily Show with Trevor Noah*	Trevor Noah	YouTube

### 3.2 Criteria of sample selection

This study established uniform requirements for data sources and program types since CTS and ETS clearly have their own program characteristics. Considering that, it was deemed necessary to make uniform requirements for each selected sample to minimize the objective differences exist between the samples of CTS and ETS for the comparison analysis. As for the screening of each sample data, the present study strictly applied the established criteria of sample selection (see Table 2).

**Table 2:** Criteria of sample selection.

Items	Criteria
Segment	Monologs
Topic theme	Life gossip and start entertainment
Content	No related ethical issues
Duration	Over 2 min for each sample
Language	American or British English for ETS and Mandarin for CTS
Quantity	100 ETS clips and 100 CTS clips
Years	2016–2022

This study mainly focused on the comparative study of humorous discourse and the rhetorical strategies of talk shows. The monolog fragment of a talk show host contains a large number of humorous words carefully designed by the talk show host—all these provided rich corpus for this study. The other segments are mainly through the interaction between the host and the guests, and the use of language is relatively small. Considering that, this study considered the monolog fragment of the talk show as the object of study.

Due to the differences between Chinese and Western societies and cultures, talk shows in China generally focus on life and entertainment gossip and do not involve sensitive political topics. In order to unify the criteria, the selected topics for this study were standardized to samples of life and entertainment gossip. At the same time, all samples were carefully selected to avoid samples involving moral and ethical issues.

This study also observed that humor requires a certain amount of padding, especially in CTS. Therefore, the selected clips had to be longer than 2 min in duration to have more valuable data. In terms of language requirements, this study unified the language used by the talk show hosts as American or British English for ETS and Mandarin for CTS. As a result, monolog samples in dialects were not selected.

In terms of sample size, this study gathered a total of 200 clips of ETS and CTS. Comrey and Lee (1992) interpreted the appropriateness of sample size for 200 is deemed reasonable. The timeframe of data sources in this study was limited to 2016 to 2022. Therefore, all data for this study’s analysis were deemed relatively new, and the topics of discussion were found to closely reflect the current interests of today’s society. Thus, the comparability of the data was further enhanced and easier to be understood.

### 3.3 Data collection and coding

As this study aimed to compare the rhetorical strategies in the humorous discourse of CTS and ETS, the original data from the archives of the selected talk shows without any intervention. In other words, this study accessed the data in its natural context. Given the focus of this study on rhetorical strategies used in humorous discourse, the humorous discourse in the monologs that made the audience laughed were marked for further analysis. The overall data collection in this study consisted of four steps.

In the first step, all samples were downloaded according to the protocol for sample selection (Table 2). The researchers downloaded each sample of ETS from its official YouTube channel or official website via online YouTube video download tool.^1^ Meanwhile, the researchers downloaded each sample of CTS from the online platform of Tencent, where the selected talk shows were broadcasted. In the second step, all downloaded videos were transcribed. For ETS, Otter.ai was used to automatically transcribe the downloaded videos. Following that, the researchers checked the ETS data for any spelling or transcription errors. For CTS, the automatic dictation function of MS Word was used to transcribe the video subtitles. Likewise, manual correction was performed to check the CTS data for any spelling or transcription errors. In the third step, humorous discourses in the transcripts were subjected to data screening. The researchers observed the downloaded videos carefully and unlined the sentences that made the audience laughed. Lastly, rhetorical strategies used to create verbal humor were coded.

The tools used for the process of data coding is Atlas.ti 22. This study used Atlas.ti 22 to code and analyze the data. Atlas.ti is a powerful data coding software that allows users to locate, code, and annotate findings in primary data, as well as weigh and evaluate their significance and visualize complex relationships (Ann and Christina, 2007). All transcribed data in this study were imported into Atlas.ti for data coding. A total of two rounds of data coding were performed.

The first round of data coding involved coding all discourses that made the audience laughed. With respect to the research questions and objectives, this study focused on parts of the discourses that produced humor and made the audience laughed. In this round of data coding, the researchers repeatedly watched and observed the video samples of CTS and ETS and coded sentences that made the audience laughed as “audiences’ laughter.” Following the completion of the first round of data coding, the software automatically counted the number of times each sample of talk show that made the audience laughed. The second round of data coding was performed according to this study’s modified list of rhetorical strategies, where all humorous discourses that made the audience laughed were screened and those that used rhetorical strategies were all coded. The process of data coding was repeated for the video samples to identify and code rhetorical strategies in the humorous discourse. Discourses that did not use rhetorical strategies were not coded in the second round of data coding. The coding procedure commenced with each researcher independently undertaking the task, subsequently followed by a comparative analysis of their respective coding outcomes. Discrepancies were deliberated upon and reconciled through discussion. Should any coding conflicts arise, the researchers sought assistance from pertinent experts in the field, ultimately reaching agreement.

### 3.4 Data analysis

This study performed the discourse analysis method to interpret and compare the rhetorical strategies used to create humor in the monologs of CTS and ETS. The results were expected to provide better understanding of the rhetorical humor in different talk shows. According to Gee (2014), discourse analysis is the study of the language used, not only for verbal expression but also to execute an action. Discourse analysis can be adopted to enhance the understanding of how language is used in communication, specifically speaking, writing, or both (Lazaraton, 2009). With respect to the research questions and objectives, two steps of data analysis were included in this study. With respect to the first objective of this study, the first step of data analysis identified the types of rhetorical strategies used to create verbal humor in the monologs of CTS and ETS. With respect to the second objective of this study, the second step of data analysis examined the similarities and differences of rhetorical strategies used to create verbal humor in CTS and ETS.

## 4 Results and discussion

### 4.1 Identification of rhetorical strategies in CTS and ETS

**Figure 1:**
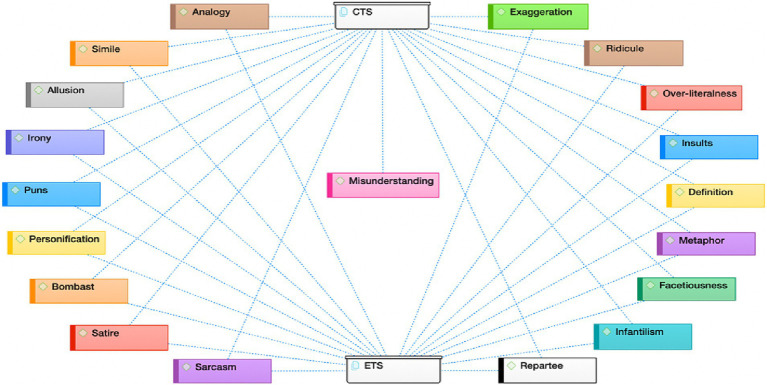
Rhetorical strategies in the monologs of CTS and ETS.

For the identification of rhetorical strategies used to create humor in the monologs of CTS and ETS, all data were coded using Atlas.ti. The obtained findings are presented in Figure 1.

For the case of CTS, rhetorical strategies of analogy, exaggeration, infantilism, ridicule, over-literalness, insults, metaphor, definition, facetiousness, bombast, repartee, sarcasm, allusion, personification, irony, puns, misunderstanding, satire, and simile were identified. This suggests the use of all 19 rhetorical strategies in the monologs of CTS. Meanwhile, as for the case of ETS, 18 rhetorical strategies were used to create verbal humor: simile, analogy, bombast, definition, repartee, exaggeration, irony, facetiousness, allusion, infantilism, metaphor, insults, personification, satire, puns, over-literalness, ridicule, and sarcasm. The line between ETS and misunderstanding was absent in the figure, suggesting that misunderstanding was not used in ETS.

**Figure 2:**
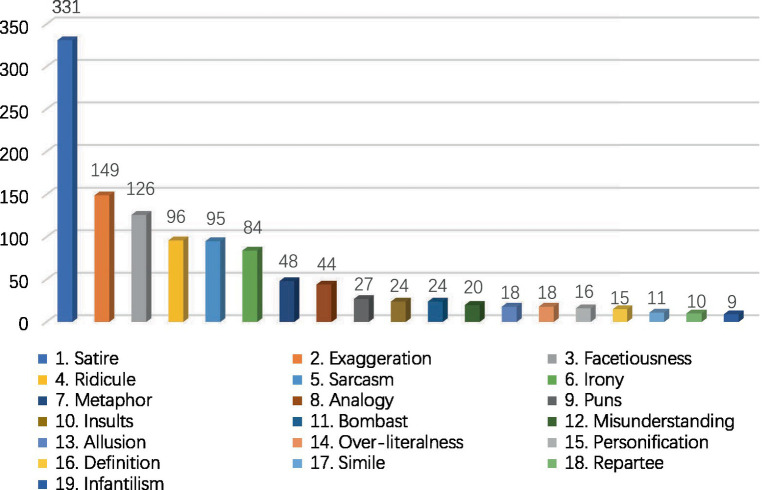
Frequency of use for each rhetorical strategy in CTS.

**Figure 3:**
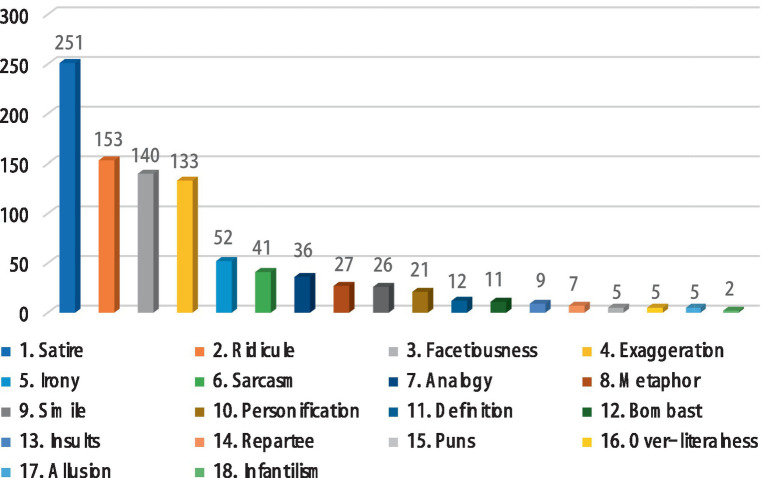
Frequency of use for each rhetorical strategy in ETS.

Following that, this study calculated the frequency of each rhetorical strategy identified in CTS (Figure 2) and ETS (Figure 3). Referring to Figures 2, a total of 1,165 rhetorical strategies were identified in the monologs of CTS. Satire (*n* = 331) was the most frequently used rhetorical strategy. Exaggeration (*n* = 149) was the second-most frequently used rhetorical strategy, followed by facetiousness (*n* = 126), ridicule (*n* = 96), sarcasm (*n* = 95), irony (*n* = 84), metaphor (*n* = 48), and analogy (*n* = 44). Puns (*n* = 27), insults (*n* = 24), bombast (*n* = 20), and the remaining rhetorical strategies recorded below 30 times. Meanwhile, repartee (*n* = 10) and infantilism (*n* = 9) were used the least.

Referring to Figure 3, a total of 936 rhetorical strategies were identified in ETS. Likewise, satire (*n* = 251) was the most frequently used rhetorical strategy. Ridicule (*n* = 153) was the second-most frequently used rhetorical strategy, followed by facetiousness (*n* = 140) and exaggeration (*n* = 133). There were also irony (*n* = 52), sarcasm (*n* = 41), analogy (*n* = 36), metaphor (*n* = 27), and simile (*n* = 26). The remaining rhetorical strategies were used less often: personification (*n* = 21), definition (*n* = 12), bombast (*n* = 11), insults (*n* = 9), and repartee (*n* = 7). Meanwhile, puns, over-literalness, and allusion were used equally (*n* = 5). Infantilism (*n* = 2) was used the least.

Based on the above findings, this study identified satire, exaggeration, facetiousness, ridicule, sarcasm, and irony as the most frequently used rhetorical strategies by CTS and ETS hosts and comedians to create verbal humor. Other rhetorical strategies were clearly used less often. Moreover, the top six rhetorical strategies in CTS (*n* = 881) and ETS (*n* = 770) accounted for 76 and 82% of the total number of rhetorical strategies used in the monologs of CTS and ETS, respectively. In other words, these rhetorical strategies were used much more often than other rhetorical strategies. Therefore, this study proceeded to analyze and discuss these six more frequently used rhetorical strategies, which are more representative both in CTS and ETS. The other rhetorical strategies in CTS and ETS account for a much smaller percentage of the total number of usages in the collected data.

Firstly, satire was the most frequently used rhetorical strategy in CTS (*n* = 331) and ETS (*n* = 251) to create verbal humor. Satire is about ridiculing individuals, organizations, and society by describing their silly behaviors (Berger, 1976). It is a position or style of humorous discourse that uses comedic devices to call attention to a particular issue and to criticize one’s shortcomings (Quintero, 2007). For example, Zhang Haozhe (CTS: Sample 86) said that he only got 57 points in the TOEFL test, but he still went to the United States to study. He proudly said:

“但是我一点都不担心啊,学语言是需要语言环境的,我刚到美国立马就学会了手语.”

(*dan shi wo yi dian dou bu dan xin a, xue yu yan shi xu yao yu yan huan jing de, wo gang dao mei guo li ma jiu xue hui le shou yu*.)

[But I’m not worried at all. Learning a language needs a language environment. I learned sign language immediately after I arrived at the United States.]

**Figure 4:**
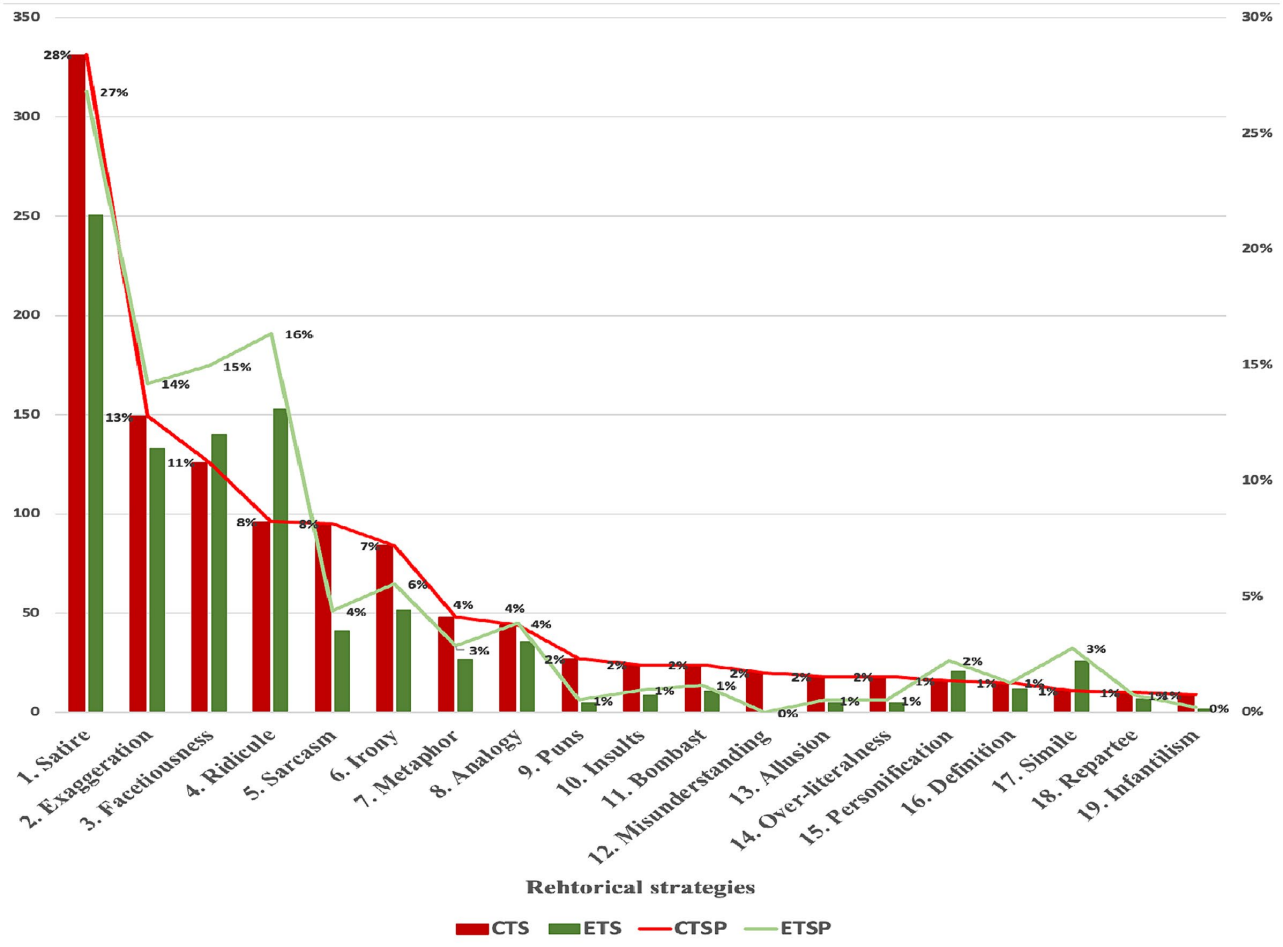
Rhetorical strategies’ percentage in CTS and ETS. CTSP denotes Chinese talk show percentage; ETSP denotes English talk show percentage.

This implies that he was not able to communicate with others in English when he was in the United States. Although he relied on body gestures to communicate with others back then, he remains proud about his experience. Zhang did not figure out the root of the problem and its solution, which stimulated the audience to imagine the image of him attempting to communicate with others. The audience felt the humor and laughed. Many satirized humorous discourses were identified in the monologs of CTS. Based on the findings in Figure 4, this study asserted the keenness of talk show hosts and comedians in CTS to use satire.

Similarly, satire was the most commonly used rhetorical strategy in ETS. Jimmy Fallon appeared to be really good at using satire to achieve verbal humor. Referring to Sample 41, Jimmy Fallon wrote his thank-you note:

“Thank you, Apple’s flagship store reopening after renovations. I hope that turning the store off and back on again fix the problem.”

This implies his dissatisfaction with the renovation of Apple’s flagship store and his concern about the quality of the Apple products. The remarks serve to satirize Apple for focusing on the decoration of their store, instead of improving the quality of their products. Referring to Sample 50, Jimmy Fallon presented another thank-you note:

“Thank you, spring weather or as we say in New York lit cigarette butts are blooming thrown into a snowbank and we are going to keep your butts.”

This satirizes certain people’s bad behavior of littering cigarette butts. It has been proposed that satire is linked to humor (Aitbaeva et al., 2018). Therefore, satire is highly useful to create verbal humor in the monologs of CTS and ETS.

Exaggeration is the second-most used rhetorical strategy in CTS, but it was the fourth-most used rhetorical strategy in ETS. Berger (1976) described that “exaggeration enhances reality and blows things up far beyond the reality of the situation” (p. 18). The case of CTS in this study revealed the use of exaggeration for 108 times to create verbal humor. Taking the example of Sample 51 of *Jin Xing Show*, Jin Xing told a story of a Chinese singer, Faye Wong, who was going to have her second baby. The paparazzi wanted to get the exclusive report, and all of them came to the hospital. However, Wong’s company did not want to leak this news to the media. As a result, they strictly cut off all possible access to Wong’s floor of the hospital. The paparazzi waited outside of the hospital for several days, and one of them found Wong after she delivered her baby. Jin Xing said:

“这个狗仔趁着保安还没有反应过来, 几乎以刘翔的速度, 跨过椅子按下快门, 拍到了现场唯一的王菲的出产房的露脸照片.”

(*zhe ge gou zai chen zhuo bao an hai mei fan ying guo lai, ji hu yi liu xiang de su du, kua guo yi zi an xia kuai men, pai dao le xian chang wei yi de wang fei de chu chan fang de lou lian zhao pian*.)

[Before the security guard could react, the paparazzi stepped over the chair and pressed the shutter, almost at the speed of Liu Xiang, and took the only photo of Faye Wong’s face in the delivery room.]

When Jin Xing described the speed of the paparazzi in order to take the photo, she described it as fast as Liu Xiang. Liu Xiang is a 110-m hurdles athlete for the Chinese men’s track and field team. On 27 August 2004, Liu Xiang broke the Olympic record for the men’s 110-m hurdles in the Athens Olympic Games with a time of 12.91 s and won the gold medal. Jin Xing exaggerated the speed of the paparazzi because only a few people would be able to move that fast like trained athletes. Exaggeration has been proved as one of the best strategies to produce humor (Tsakona, 2009), which explains its frequent use in CTS by talk show hosts and comedians in their monologs.

Likewise, exaggeration is one of the favorite rhetorical strategies in ETS. Referring to Sample 10 (“*Ellen Reviews Four Fun Facts You Never Know About Cher*” on 15 September 2018), Ellen DeGeneres talked about Cher’s song that was released in 1998, entitled “Believe.” Ellen DeGeneres said:

“Anyway, that song was released in 1998. And I cry every single time I hear it because that was the year my sitcom got canceled.”

This discourse made the audience laughed. Ellen DeGeneres’s exaggeration showed her liking Cher’s songs and that one of her songs made Ellen DeGeneres recalled certain memories. However, she said that she would cry every time she heard Believe, which was an exaggeration. Berger (1976) viewed that exaggeration can also “be reversed, leading to humorous understatement” (p. 18). Another example from Sample 97 on the topic of “*Four Times Trevor Was Starstruck*,” Trevor Noah described a situation during the Oscars when he pushed Jay-Z to go through the surrounded fans. The security man saw and approached him, which made him scared: “And this guy just he came out he was going to break me. It was like slow motion.” The audience laughed because the talk show host exaggerated the reality of the security man’s movement speed.

Facetiousness was identified as the third-most rhetorical strategy used to create verbal humor in both CTS (*n* = 126) and ETS (*n* = 140). Berger (1976) described facetiousness as joking or frivolous, non-serious use of language and attitude by a character. Referring to Sample 84, Tong Monan’s girlfriend complained about his shortage:

“我就纳闷为什么人的优点往往鱼和熊掌不可兼得,但是缺点竟然可以一点排异反应都没有, 很容易就做到五毒俱全啊!”

(*wo jiu na men wei shi me ren de you dian wang wang yu he xiong zhang bu ke jian de, dan shi que dian jing ran ke yi yi dian pai yi fan ying dou mei you, hen rong yi jiu zuo dao wu du ju quan a!*)

[I wonder why people’s advantages are often incompatible with one another, but the disadvantages are easy to achieve!]

The above sentence in Mandarin brings humor due to the combination of different meanings of words that they do not match one another. It sounds ridiculous with such a mass of words used, which made the audience laughed.

As for the case of ETS, this study found Ellen DeGeneres is very good at using facetiousness to create verbal humor. Referring to Sample 16 on “*Ellen Is Hurt!*,” at the beginning of the show, Ellen DeGeneres came out with crutches and said:

“Hello! All right, I have some good news, I have some bad news. The good news is, I do not need these. Bad news is I hurt my neck.”

When the audience saw her walking out with crutches, they thought she injured her leg, but they did not expect that she injured her neck. Although she was injured, she told the audience with such frivolous language. The rhetorical strategy of facetiousness was used here to make the whole studio lively.

Although the other talk shows showed less common use of facetiousness, the rhetorical strategy remains powerful in creating laughter. For instance, referring to Sample 65, Jimmy Fallon said:

“Thank you, Santa Claus, for festively testing the limits of breaking and entering laws.”

It is illegal to break and enter people’s house without permission, but Jimmy Fallon applied this law on a fairy tale character. He applied a non-serious use of language on the behavior of Santa Claus. Although facetiousness is an easy way to make funny, being facetious has a drawback of being easily misunderstood; one must somehow make it clear to the audience that one is being ironic (Berger, 1976). To date, the rhetorical strategy of facetiousness has remained under-researched.

Ridicule was identified as the fourth-most used rhetorical strategy in CTS (*n* = 96) and the second-most used rhetorical strategy in ETS (*n* = 153). Ridicule involves “making fun” and casting contemptuous laughter at someone or something (Berger, 1976). Talk show hosts and comedians make fun at someone or something through this strategy to create verbal humor. Referring to Sample 9, Wang Zijian described a girl who complained about her boyfriend’s lack of study motivation, and that her parents would not agree to their relationship. Surprisingly, the boyfriend answered that his parents could send him to the United States or Japan to further his studies because they have houses in these two countries. The girl was very happy. Wang Zijian ridiculed the girl for her innocence and vain psychology:

“那这个女生也是头发长见识短是吧, 日本跟美国的房子哪有上海的房子贵呀!”

(*na zhe ge nü sheng ye shi tou fa zhang jian shi duan shi ba, ri ben gen mei guo de fang zi na you shang hai de fang zi gui ya!*)

[Then the girl has long hair and less knowledge, right? How can the houses in Japan and the United States be more expensive than the houses in Shanghai!]

Meanwhile, as for the case of ETS, Trevor Noah appeared to favor the rhetorical strategy of ridicule to create verbal humor. Referring to the sample of “*Trevor’s Unexpected Ride to Work*,” he shared one of his interesting experiences of going to work. One day, when he was on the way to the talk show, someone in one of those sanitation trucks was sweeping the streets. They met each other at a narrow road, and then he jumped into the sanitation truck and swept the streets together with the driver. He was supposed to come straight to work, but then he drove around New York and swept the streets with the other person. His description of his ridiculous behavior made the audience laughed. This particular rhetorical strategy is often employed by talk show hosts to mock someone’s ridiculous activities.

Sarcasm was identified as the fifth-most used rhetorical strategy in CTS (*n* = 96) and sixth-most used rhetorical strategy in ETS (*n* = 41). Sarcasm means “tearing the flesh” or “biting the lips in rage” (Berger, 1976, p. 38) and refers to the use of language that is contemptuous, mocking, and wounding. As previously mentioned, Jin Xing is famous for her bitter language in her talk show program. Referring to Sample 40, Jin Xing mentioned her mother’s keenness of introducing partners to unmarried people, and her mother once said to a single man when she was persuading him to go on a date:

“哎呀, 我跟你说了别想那么多了就你这个模样啊, 人家观众能看上你都算社会没把你给抛弃了, 就不错了.”

(*ai ya, wo gen ni shuo le bie xiang na me duo le jiu ni zhe ge mo yang a, ren jia guan zhong neng kan shang ni dou suan she hui mei ba ni gei pao qi le, jiu bu cuo le.*)

[Oh, I told you not to think so much. If others like you for your looks, you should consider that society did not abandon you. It is good enough.]

She demonstrated the use of sarcasm to satirize the man who is not good looking but picky about who they meet on a date. Many Chinese talk show hosts and comedians in CTS prefer the use of sarcasm on their own or others’ appearance to create verbal humor. For instance, referring to Sample 72, Xu Zhisheng said that, after he gained a bit of fame, a company invited him to sell facial masks on live stream. He asked the manager:

“你到底想通过我这张脸来表现这个面膜的什么作用? 副作用吗?”

(*ni dao di xiang tong guo wo zhe zhang lian lai biao xian zhe ge mian mo de shen me zuo yong? Fu zuo yong ma?*)

[What exactly do you want to show through this face of mine about the effects of this mask? The side effects?]

The audience laughed out loud at his remark, as live streams typically involve beautiful or handsome hosts to sell products, such as facial masks. In this humorous discourse, Xu Zhisheng expressed that he would be more likely to show the side effects of the facial masks. He was sarcastic about his own looks and thought that selling the facial masks would be an impossible task for him.

As previously mentioned, sarcasm involves using bitter language to satirize certain people and things. Referring to Sample 98, Trevor Noah described a couple who killed an animal for fun and posed for an intimate photo next to its carcass. The photo of an African trophy hunt has sparked outrage across the country. Trevor Noah then commented:

“Yeah, like a family funeral. Suddenly, just like two lions popped out and started humping at your dad’s coffin, just like you would not be happy with that.”

Trevor Noah’s bitter language criticized and satirized the behavior of the couple. He entertained the audience and promoted wildlife protection and respect for the nature. Apart from creating humor, according to Tao (2007), in a post-totalitarian society, sarcasm may be “the only viable means of resistance for young generations” (p. 217).

Irony involves saying one thing but meaning the opposite (Berger, 1976). In other words, it describes literal words that are sometimes the inverse of the intended meaning (Rucynski, 2022). Ortega (2013) claimed the frequent co-existence of irony and humor in speech. The current study identified 95 cases of irony in CTS and 52 cases of irony in ETS, suggesting its common use to create verbal humor in the monologs. For instance, referring to Sample 19, Wang Zijian said that Jianguo’s father often praises him by posting on WeChat Moments:

“你们知道我儿子吗? 我儿子是全校老师最佩服的一个孩子. 老师经常跟我儿子说, 王建国你回家吧, 没人教得了你.”

(*ni men zhi dao wo er zi ma? Wo er zi shi quan xiao lao shi zui pei fu de yi ge hai zi. Lao shi jing chang gen wo er zi shuo, wang jian guo ni hui jia ba, mei ren jiao de le ni*.)

[Do you know my son? My son is the most admired child in the whole school. The teacher often tells my son, Wang Jianguo, go home, no one can teach you.]”

The word “佩服” (*pei fu*) is a form of irony; it means “admire,” but Jianguo’s father meant “hate” or “dislike” because Jianguo was considered too stupid to learn, as indicated by his teacher. Irony was also adopted in ETS. Referring to Sample 70, Trevor Noah told a story that he heard—it was about a man who fell in love with Atlanta due to an unexpected reason. He replied:

“What happened? Oh, wow. Oh man. So, you go to Atlanta. The recession happens. So now you are stuck in Atlanta. That is so dope. I feel like I should make a TV show about you, man. It’s like a fun story. It’s like I ended up in Atlanta.”

In this discourse, Trevor Noah used the sentence, “That is so dope,” which appeared as if he was praising the man, but ironically, it was unfortunate for him at that time. In fact, the man got lost and had to stay there for a long time instead. Most importantly, irony means satirizing something or someone’s behavior in a reverse expression. As a result, irony is seen as a pragmatic phenomenon supported by indicators, making it possible to provide an explanation that transcends specific contexts in which it occurs (Ortega, 2013).

Apart from the six most frequently used rhetorical strategies in CTS and ETS, talk show hosts and comedians also adopted other rhetorical strategies to produce good humor effects although these strategies were used less often. For instance, metaphor is a figure of speech containing an implied comparison (Tirrell, 1991). Weaver (2015) regarded metaphor as an elementary rhetorical strategy to create humor. Piata (2016) shared the same view on metaphor. Referring to Sample 27, Wang Zijian and Wang Jianguo encountered a mother and her daughter 1 day. The mother pointed at Wang Jianguo’s stomach; she used a metaphor by describing his large stomach as a watermelon to prevent her daughter from eating watermelon seeds:

“你看那个叔叔就是因为把西瓜子吃到肚子里面去了, 你看他那个肚子里面长西瓜了吧!”

(*ni kan na ge shu jiu shi yin wei ba xi gua zi chi dao du zi li mian qu le, ni kan ta na ge du zi li mian zhang xi gua le ba!*)

[Look at that uncle; because he ate watermelon seeds in his stomach, so his stomach is growing watermelon!]

Likewise, referring to Sample 14, Ellen DeGeneres described her hair after dyeing it: “And I had the pride flag on my head.” She implied that her hair had the same color as a flag, which was humorous and stimulated the imagination of the audience. Additionally, referring to Sample 51, Jimmy Fallon said, “Thank you marbles for being rocks that just dropped acid,” suggesting that the shape of the marbles is the same as the shape of the rock acid.

All rhetorical strategies serve as a powerful tool for talk show hosts and comedians to create verbal humor. The discussed findings clearly demonstrated the diversity of rhetorical strategies used to create verbal humor in CTS and ETS. Satire, exaggeration, facetiousness, ridicule, sarcasm, and irony were found to be the most frequently used rhetorical strategies in CTS and ETS, which accounted for more than half of the total number of rhetorical strategies identified. The use of other rhetorical strategies was found to be relatively less common, especially the use of repartee and infantilism. Moreover, the rhetorical strategy of misunderstanding was not found in ETS. The finding also addressed Mulyadi et al.’s (2021) claim that verbal humor is often achieved via different types of rhetorical strategies in certain situations. This study observed the willingness of talk show hosts and comedians to use rhetorical strategies to achieve good humor and entertainment effects (Tianli et al., 2022), which may explain why both CTS and ETS in this study employed numerous rhetorical strategies. According to Weaver (2012), humor offers a variety of ideological or discursive effects through rhetoric, while the dynamics of the rhetorical triangle may influence the humorous meanings generated by jokes. This study supported this particular argumentation; the described examples in this subsection clearly showed the skills of talk show hosts in both CTS and ETS in using various rhetorical strategies to achieve humor and create a more relaxing and interesting show for the audience. Based on the obtained findings, the current study proved the important roles of rhetorical strategies for talk show hosts and comedians to create verbal humor and enhance their humorous language in monologs (Wang, 2019; Hu, 2020).

### 4.2 Similarities and differences of rhetorical strategies

This subsection presents the similarities and differences of the identified rhetorical strategies in CTS and ETS. After careful data coding and repeated data checking, the study identified a total number of 2,101 rhetorical strategies in the collected data, which consisted of 1,165 rhetorical strategies in the CTS data and 936 rhetorical strategies in the ETS data. Overall, it was evident that both CTS and ETS used rhetorical strategies to create verbal humor. The number of identified rhetorical strategies in CTS was higher than the number of identified rhetorical strategies in ETS, suggesting the higher likelihood of talk show hosts and comedians of CTS adopting rhetoric to produce humor. A clear comparison of the use of each rhetorical strategy for CTS and ETS is presented in Figure 4.

Referring to Figure 4, the present study observed an unevenly distributed rhetorical strategies in talk shows. All identified rhetorical strategies were used at different frequencies. As for the case of CTS, 19 rhetorical strategies were used. Meanwhile, only 18 rhetorical strategies, except for misunderstanding, were used for the case of ETS. In general, this study found numerous similarities and differences in the use of these rhetorical strategies in CTS and ETS.

Firstly, based on the percentage of use of various rhetorical strategies, the top six rhetorical strategies used to create verbal humor in CTS and ETS were satire, exaggeration, facetiousness, ridicule, sarcasm, and irony. The other rhetorical strategies were used less frequently. Satire was found to be the most used rhetorical strategy in both CTS (28%) and ETS (27%). In other words, talk show hosts and comedians on both talk shows preferred to use satire. The subsequent most used rhetorical strategies in CTS were exaggeration (13%), facetiousness (11%), and ridicule (8%). On the contrary, the subsequent most used rhetorical strategies in ETS were ridicule (16%), facetiousness (15%), and exaggeration (14%). There were no significant differences in the number of usages for facetiousness and exaggeration between CTS and ETS, but the percentage of usages for them in ETS was higher than that in CTS, suggesting that ETS used facetiousness and exaggeration more often to create verbal humor. Moreover, the most obvious contrast between CTS and ETS is that the percentage of ridicule strategy is much higher in ETS (16%) than in CTS (8%), which indicates that the ETS hosts prefer to use ridicule to create verbal humor in their monologs than CTS hosts.

Secondly, most of the remaining rhetorical strategies were used more often in CTS than ETS, such as sarcasm, repartee, puns, over-literalness, misunderstanding, metaphor, irony, insults, infantilism, definition, bombast, analogy, and allusion. More surprisingly, ETS did not apply the rhetorical strategy of misunderstanding, but this study found 20 cases of misunderstanding (2%) used in CTS. Besides that, the percentage of usage for sarcasm, metaphor, irony, insults, and bombast in ETS was almost half or slightly more than half of the number of cases of usage in CTS. Meanwhile, the percentage of usage for puns, over-literalness, infantilism, and allusion in CTS was far higher than in ETS. However, the percentage of usage for repartee (1%), definition (1%), and analogy (4%) was the same between CTS and ETS.

Thirdly, simile and personification were used more often in ETS than in CTS. The use of simile was identified as accounting for 3% of ETS and 1% of CTS. Besides that, the use of personification accounted for 2% in ETS and 1% in CTS.

Both CTS and ETS used rhetorical strategies to create verbal humor, but the use of these strategies was unevenly distributed. Besides that, both talk shows used satire, facetiousness, ridicule, and exaggeration more frequently than other rhetorical strategies. The difference between CTS and ETS was that most of the rhetorical strategies used in CTS were used more often than in ETS. Moreover, CTS recorded the use of misunderstanding rhetorical strategy, but none was found in ETS.

Numerous prior studies (Wang, 2019; Hu, 2020; Jiang, 2021) identified many types of rhetorical strategies in CTS and ETS, which showed the significant role of rhetorical strategies in producing humor. Therefore, mastering the rhetorical use of language is an important skill for a talk show host or comedian to present humorous discourses (Wang, 2019). This study demonstrated the preference of talk show hosts and comedians of CTS to use rhetoric for their humorous discourse than those of ETS. This may be attributed to their view on the use of rhetorical language to improve artistic expression (Wang, 2019). Although Jiang (2021) and Hu (2020) identified the use of several rhetorical strategies in CTS, such as exaggeration, metaphor, and allusion, these prior studies did not fully explore other types of rhetorical strategies. As for the current study, 19 rhetorical strategies were identified in the humorous discourse of CTS, which provided a good reference point for future research. As for the case of ETS, this study clearly demonstrated the skills of talk show hosts and comedians of ETS in using rhetorical strategies to create verbal humor despite their less frequent usage as compared to those of CTS.

Following that, this study examined the percentages of the total types of rhetorical strategies to produce humor. Referring to Table 3, the verbal humor in the monologs of CTS was realized through 82% of rhetorical strategies and 18% of non-rhetorical strategies, while the verbal humor in the monologs of ETS was achieved through 72% of rhetorical strategies and 28% of non-rhetorical strategies. This confirmed that most of the humorous discourses in talk shows employed rhetorical strategies to create verbal humor. Moreover, the percentage of rhetorical verbal humor in CTS was slightly higher than in ETS. Consequently, the non-rhetorical verbal humor in ETS was higher than in CTS.

**Table 3:** Percentages of rhetorical strategies to create verbal humor in CTS and ETS.

Verbal humor in monologs	CTS	ETS
Verbal humor produced by RSs	763	609
Verbal humor produced by other methods	162	236
Total verbal humor produced in talk show	925	845
Percentage of rhetorical humor	82%	72%
Percentage of no-rhetorical humor	18%	28%

RSs denotes rhetorical strategies.

The results of Table 3 indicate that the talk show hosts or comedians rely very much on rhetorical strategies to produce humorous discourses. Overall, rhetorical strategies used in CTS and ETS not only shared several similarities but also had differences in the aspects of the adopted types and usage to create verbal humor. More importantly, rhetorical strategies accounted for a large proportion of the talk shows’ verbal humor production, which was neglected by most previous studies on talk shows. Even though previous studies have emphasized the significance of the relationship between rhetorical strategies and humorous discourses, such as studies of Rutter (2001) and Heidari-Shahreza (2017). As CTS and ETS involve different languages, the rhetorical strategies they use are influenced by their different linguistic, cultural, and social factors (Rochmawati, 2017; Dedace et al., 2023). In terms of its relevance to personal experience, the language one uses and listens to in everyday life differs significantly (Lyons et al., 2010). Moreover, people develop a distinct style of speech as a result of their various social backgrounds and experiences (Lizardo, 2021). Talk show hosts and comedians come from different countries and experience different social contexts and personal experiences, which contribute to the similarities and differences of rhetorical strategies in CTS and ETS.

## 5 Conclusion

This study found that CTS recorded a total of 19 rhetorical strategies to create verbal humor in the monolog discourse. Meanwhile, this study found that ETS employed similar rhetorical strategies as CTS, except for the rhetorical strategy of misunderstanding. The most frequently used rhetorical strategies in both CTS and ETS were satire, exaggeration, ridicule, and facetiousness. With respect to the second research question, this study first compared the identified rhetorical strategies in CTS and ETS. Based on the findings, this study observed higher number of rhetorical strategies used in CTS (*n* = 1,165) than ETS (*n* = 935). Specifically, the most frequently used rhetorical strategies in both talk shows were almost similar (e.g., satire, exaggeration, facetiousness, and ridicule), but the frequencies of usage of these various rhetorical strategies in both talk shows were slightly different. Besides that, CTS employed the majority of the remaining rhetorical strategies more frequently than ETS. Interestingly, misunderstanding occurred 20 times in CTS but was not found in ETS. Meanwhile, simile and personification were used more often in ETS. Moreover, this study found that the probability of achieving humor through rhetorical strategies accounted for 82 and 72%, respectively, which indicated rhetorical strategies as a common means of creating verbal humor in the monologs of CTS and ETS. Overall, the comparison of the types of rhetorical strategies in the humorous discourse of CTS and ETS revealed the existence of both similarities and differences.

This study shed light on the importance of rhetorical strategies to create verbal humor. The contribution of this study is threefold. Firstly, this study identified all rhetorical strategies used to create verbal humor in the monologs of CTS and ETS, which enhanced the overall understanding of the humorous discourse of talk shows in different languages. Secondly, the identification of similarities and differences in rhetorical strategies in the humorous discourse of CTS and ETS in this study would benefit the audience, helping them to grasp the characteristics of rhetorical strategies used in different types of talk shows. Therefore, this would encourage and help the audience to favorably perceive and appreciate the humor of talk shows in different languages. Finally, the current study presented several noteworthy contributions to the methodology of verbal humor discourse analysis. This study proposed an enriched list of rhetorical strategies to create verbal humor based on Berger’s humor rhetorical strategies. It also provided detailed examples of humorous discourses in different talk shows, which were expected to benefit future research as significant references.

Despite of that, this study encountered some limitations. For instance, this study only focused on examining and comparing rhetorical strategies to create verbal humor in the monologs of CTS and ETS. The other sections of the talk show program, such as guest or celebrity interview section, were not included in the study. Consequently, the use of rhetorical strategies in the entire talk show program was underexploited. Additionally, the variation between the different language talk shows would, to some extent, affect the occurrence of the rhetorical strategies’ adoption, such as the different styles of the talk show hosts and the different talk show segment arrangement sequence, which is not investigated by the present study.

Addressing the identified limitations of the current study, this section presents some recommendations for future research. Firstly, it is recommended for future research to examine rhetorical strategies used in other sections of talk shows or other types of humorous discourses of talk shows, as well as to examine what and how the rhetorical strategies are applied in the interactions of talk show hosts and comedians with the guests and audience. In addition, it is recommended for future research to explore the talk show hosts’ or comedians’ attitudes and opinions in order to obtain more comprehensive evidence on the choice of rhetorical strategies in talk shows of different languages. Moreover, the factors that impact on the rhetorical strategies use in different languages or context could be future examined.

## Data availability statement

The original contributions presented in the study are included in the article/supplementary materials, further inquiries can be directed to the corresponding author/s.

## Author contributions

ZT: Formal analysis, Investigation, Methodology, Software, Writing – original draft, Writing – review & editing. SC: Data curation, Resources, Supervision, Validation, Writing – review & editing.

## Footnotes

^1^ https://zh.savefrom.net/7/
